# Influence of the implementation of strengths-based nursing and healthcare on early childhood nurses’ competencies: a mixed-method study

**DOI:** 10.1186/s12913-022-08955-7

**Published:** 2022-12-22

**Authors:** Camille Thentz, Christine Durgnat-Sciboz, Sylvie Macé, Marie-Catherine Béguin, Vincent Falcy, Elisabeth Schobinger, Gora Da Rocha

**Affiliations:** 1 Centre de référence pour les infirmières Petite enfance, Association Vaudoise d’Aide et de Soins À Domicile, 1014 Lausanne, Switzerland; 2 School of Health Sciences, HES-SO University of Applied Sciences and Arts Western Switzerland, 1206 Geneva, Switzerland; 3 Service du Développement des Pratiques Professionnelles, Association Vaudoise d’Aide et de Soins À Domicile, 1014 Lausanne, Switzerland; 4 School of Health Sciences, HES-SO University of Applied Sciences and Arts Western Switzerland, 1011 Lausanne, Switzerland

**Keywords:** Pre/post intervention design, Strengths-based approach, Early childhood nurse, Competency, Mixed-methods

## Abstract

**Background:**

The scope of practice for nurses caring for families has evolved to meet the challenges presented by societal changes and increasing needs. In 2015, early childhood nurses from a Swiss region decided to implement a new model of care to guide their practice. The aim of this study was to explore the changes to early childhood nurses’ practices following the implementation of the strengths-based nursing and healthcare (SBNH) approach to care.

**Methods:**

This study of early childhood nurses’ (*N* = 61) practices used a pre-post intervention design and a mixed-method approach. Nurses’ competencies and changes in practice were measured using the Nurse Competence Scale (NCS). The quantitative data were analysed using descriptive statistics, Kruskal Wallis tests and logistic regression. Thematic analysis was used to derive themes from the qualitative data.

**Results:**

After the intervention, frequency of competency use increased in all domains of the NCS except the “teaching-coaching” domain; perceived levels of competencies also increased in all areas except “helping role” and “diagnostic functions”. Age and length of employment at the current post hindered improvement in the “teaching-coaching” competency. Interviews revealed themes related to the implementation process: “adaptability”, “implementation process”, “ambivalence” and “engagement to change”. Other themes were related to practice changes: “developing a disciplinary identity”, “path with families” and “strengths”.

**Conclusion:**

This study showed that the use of perceived competencies changed over time after the introduction of SBNH into practice. Nurses questioned and adapted their routines based on SBNH. Nurse’s vision of care also changed; they felt that their care was congruent with their values. For families, this approach allowed a change of vision with a resource-centred approach. Implementation of models of care such as the SBNH in the early childhood context is innovative, as little research in the literature addresses the early childhood community home-visiting context is still modest. This research underlines the added value of this approach on early childhood nurses’ competencies.

## Contributions to the literature

- This study identified the added value of the implementation of the SBNH on early childhood nurses’ perceived competencies.
- The I-PARISH framework was used to guide the implementation of a strengths-based nursing and healthcare approach in an early childhood context.
- Strengths-based nursing and healthcare is a promising approach to changing nursing practice and valuing family strengths.

## Background

The scope of practice and role of nurses working with families has evolved to meet the challenges presented by societal changes and expanding needs. Indeed, the complexity and vulnerability of family presentations (e.g., poor social support, low socioeconomic status) has increased [1, 2], leading to greater complexity in the role of early childhood nurses’ (ECN) [1, 3]. ECN traditionally focused on following the child’s growth and development, but their role has developed to include parental mental health assessment and support [1]. This broader focus requires a shift in practice from a problem-oriented model to a strengths-based model [3, 4].

In 2015, a Swiss community home-visiting institution launched a project to enhance the knowledge and nursing skills of ECN, drawing on evidence-based practice recommendations [5, 6]. The focus of this implementation project was to identify and to improve ECN’s perceived competencies. The aims were 1) to update knowledge about the fundamental nursing theories and concept that underpin nursing practice and 2) to agree to and share a person-centered care approach guiding ECN’s practice: strengths-based nursing and healthcare (SBNH) [7].

In the French-speaking part of Switzerland, ECN’s role is defined by a framework as promoting a favourable family and social environment to the optimal development of children from birth to the age of four and preventing psycho-affective disorders, illnesses and accidents occurring early in life [8]. ECN recognize, support and strengthen parents’ capacities to meet their children’s needs through free home visits, group meetings and/or phone consultations, where they offer listening, support, advice, and guidance to promote children’s development [8, 9]. This is in accordance with international definitions that promote the use of strengths-based approaches [10]. Since the introduction of SBNH, the focus has been on supporting families in identifying and empowering their strengths. SBNH is defined as “a new way of thinking in nursing philosophy that shifts from a deficit, reductionist lens to a strengths-based, holistic lens” [11].

Conceptual or theoretical models are intended to guide nursing practice and research to advance and share nursing discipline-specific knowledge, allowing the profession to contribute to the betterment of humanity [12].. The use of care models in ECN’s clinical practice is still minimal [5]. Some middle-range theories have been used, such as Orem’s self-care deficit theory or Newman’s health as expanding consciousness theory [5]. Furthermore, ECN’s practice also draws on knowledge of other discipline such as psychology (e.g., Bowlby’s attachment theory) [5]. However, the literature review in this study did not identify any research addressing the use of the SBNH in the context of care for young children.

SBNH shifts the focus from deficits, problems and weakness to using strengths and resources to cope with problems and overcome weaknesses [13–16]. This approach has the potential to become an important impetus of change in nursing, shifting practice away from the deficit approach used in the past, which provided quick but expensive and less effective solutions [7, 13]. Furthermore, health systems are increasingly moving towards a health promotion vision in which individuals and communities assume greater control and responsibility for their own health and care decisions [7, 13, 14].

This approach is already used internationally and in other contexts (e.g. palliative care, supervision of nursing trainees, child welfare) [17–23]. According to Gao et al. (2018), it is important to use a vocabulary based on the patient’s strengths when assessing health risks [17]. A 2019 literature review highlighted the benefits of using this approach in palliative care, allowing ethical and relational care for the patient and their family [18]. This approach is also useful in the supervision of nursing trainees. It allowed student empowerment, collaborative learning and mutual growth [19]. In child welfare, this approach led to a greater engagement of parent in changing their behaviours and a facilitated collaboration [20, 23].

In the paediatric context, SBNH has shown good results in the care of children with autism and their families [24]. It increased their participation in the everyday life activities and promoted family-coach collaboration [25]. Used with interdisciplinary practice, the approach has confirmed its capacity to focus on the strengths of the children and their families and to engage them in the process of care [25]. A study of adolescents with type 1 diabetes (2019) showed increased adherence to protocols and improved relationships between healthcare providers and families when providers focused on positives adolescent behaviours. It also showed decreases in the perceived level of burden reported by parents [26]. A study from Toback et al. (2016) noted that a simple strengths-based intervention for adolescents admitted to a psychiatric hospital resulted in improvements in self-esteem and self-efficacy [27]. Parents who received a strength-based intervention reported higher confidence in their parenting role [28]. Based on the literature, adopting a new vision of care through the use of SBNH could make a real contribution to guiding the practice of ECN. We concluded that strengthening and supporting the resources of parents and families would positively influence the relationship with parents, thus supporting the development and health of the child. Furthermore, SBNH provides nurses the opportunity to develop their competencies [13]. SBNH is both a philosophy and value-driven approach that can guide clinicians in their competencies [16]. Eight core values rooted in principles of person/family centered care, guide nursing action, promoting empowerment, self-efficacy and hope. At all levels of care, from primary care of healthy patients to intensive care of unconscious patients, SBNH reaffirms the goals of nursing to promote health, facilitate healing, and relieve suffering by creating environments that work with and enhance patients’ health capacities and innate healing mechanisms [15, 16].

Although, the added value of SBNH has been demonstrated, its implementation remains inconsistent [21, 22]. One study in the early childhood context found that nurses needed to understand the potential benefit of the approach to adhere to it [29] and highlighted that transitioning to a different model of care is challenging for nurses [28, 29]. More research is needed to better understand the practice changes induced by the implementation of a strengths-based model in early childhood context and which factors influence the implementation [23].

The objective of the study was to explore the evolution of ECN’s practice (in the following competencies: helping-role, teaching-coaching, diagnostic functions, managing situations, therapeutic interventions, ensuring quality and work role) following the implementation of SBNH [30].

## Methods

### Study design

This longitudinal pre-post intervention study used a mixed-methods design. In order to gain a broader understanding of the perception of the intervention from all of the ECNs from this specific home care institution, all (*N* = 61) were invited to participate in the study.

Specific objectives were

- to assess the perceived change in the degree of nursing competence and the frequency of use of the different dimensions of SBNH in their practice before and after implementation (quantitative data),
- to explore ECN’s experiences on the implementation process of the SBNH approach qualitative data), and
- to explore practice changes after the implementation of SBNH (qualitative data).

### Implementation strategy: implementation facilitation

**Fig. 1:**
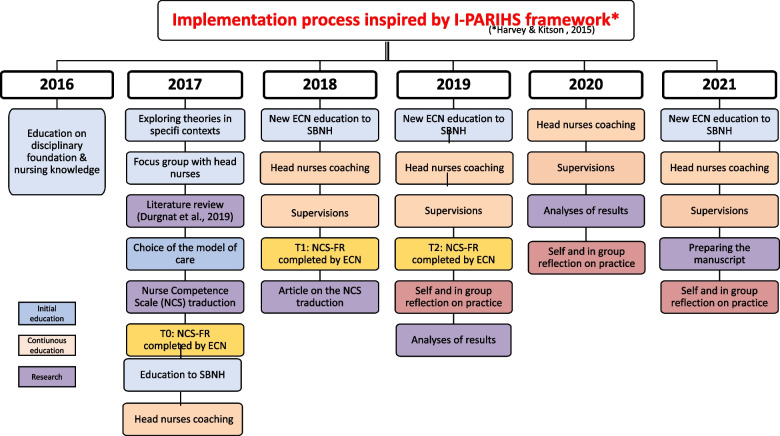
Implementation Process inspired by I-PARIHS Framework

Implementation of an intervention is considered successful when the identified goals have been achieved and the intervention has been adopted and institutionalized [31]. Involvement of the stakeholders in the project facilitates the adoption of the intervention [31]. This study used the Integrated Promoting Action on Research Implementation in Health Services (I-PARIHS) framework to guide the implementation process. This framework highlights that successful implementation of innovations depends on the inner and outer context, the recipients and the characteristic of the innovations itself. Facilitation is viewed as the “active ingredient”, with designated facilitators (internal or external) activating implementation by assessing and guiding the recipients (ECN) of the intervention (SBNH) through their contexts (early childhood home care). For this study, we used an external facilitator, an expert in nursing theories and SBNH. Figure 1 presents the SBNH implementation process.

### Intervention

Intervention was reported according to the Standards for Reporting Implementation Studies (StaRI) guideline [32]. Head nurses were initially trained in the theory during two half-day sessions. During implementation phase, the head nurses received coaching in group three times a year from an expert trainer, a professor and researcher of the strength-based nursing approach (See Fig. 1). The coaching allowed them to reflect on the changes in their leadership that had occurred while using the SBNH approach and their support for ECN during its integration. These steps were documented to create a guide on how to deploy SBNH in ECN’s practice.

After the head nurses were well prepared, all ECN attended a one-day training in November 2017. They then took part in workshops, either in small, regional teams with their head nurses or in individual interviews, to reflect on and experiment with concrete strategies for the implementation of the approach. Subsequently, ECN received 3 hours of supervised training from the expert trainer eight times per year.

ECN’s practice is based on Gottlieb’s book, *Strengths-based Nursing Care* [7]. With the support of the head nurses, ECN documented their approach and developed the SBNH concepts in their practice with families. In addition, a scientific colloquium in 2018 provided an opportunity to present the approach to Professor Gottlieb, who welcomed the project and encouraged its deployment.

### Data collection

The recruitment process started in November 2017. The call for participation was sent to all ECN in the region. Online questionnaires were sent at three time points: before the intervention (T0), 6 months after intervention (T1) and 18 months after intervention (T2). Nurses were allowed 1 hour of working time to fill out each questionnaire. Questionnaires were sent via email to increase the participation rate. Reminders were sent out 2 weeks apparat and after one round, participants were considered lost to follow-up. Nurses were given the opportunity to ask the study investigators questions anonymously.

### Instruments

The following sociodemographic data were collected: age, nursing degree, work experience in hospital, experience at actual post and work rate.

### Perceived competencies and level of expertise

To assess nurses’ perceived competencies and level of expertise, the Nurse Competence Scale (NCS) was used. Nurses self-assessed their level of competency and the frequency of their actions regarding seven domains [33]. This 73-item scale was translated and culturally adapted into French according to Wild et al. (2005) by our research team [6, 34] (see Table 1).

**Table 1:** Translation steps

*Steps*	*Activity*
*Preparation*	*In month Year the author of the questionnaire and the journal edito where the questionnaire was published were contacted to obtain consent for translation* *Developing the role of every research member involved in the translation*
*Forward translation*	*Two research members, who are bilingual, translated independently the questionnaire into French, and then compared their version*
*Reconciliation*	*Research members reconciled the two versions in order to resolve the discrepancies and obtain one final French version*
*Backward translation*	*Back translation into English by two other bilingual researchers who did not knew the original version*
*Back Translation review*	*Research members reconciled the two English versions obtained in order to resolve the discrepancies and the compared the final backward version with the original version*
*Harmonization*	*After final decision by the research members, an external expert with a master’s degree in nursing with an excellent level of English was asked to clarify whether the items seemed to fit.*
*Cognitive debriefing*	*The new version was tested with French speaking nurses working in the institution, and also reviewed by the headnurses. Based on those feedbacks, research members finalized the French version*
*Review of Cognitive Debriefing Results and Finalization*	*Based on the feedbacks obtained in the cognitive debriefing, research members finalized the French version.*
*Proofreading*	*The proofreading was done by a person external to the research group but working in the institution*
*Final report*	*The report with all the steps was addressed and approved by the author of the original version*

The NCS has good internal consistency (α = 0.97) [6]. The following seven domains compose the scale: “helping-role”, “teaching-coaching”, “diagnostic functions”, “managing situations”, “therapeutic interventions”, “ensuring quality” and “work role”. The perceived level of competency in each item is assessed using a visual analogic scale, (ranging from 0 = lowest level of competency to 100 = highest level of competency) and the frequency of action is assessed using a Likert scale (ranging from 0 = not applicable to 3 = very often) [6, 33].

### Vision of care

As a new philosophy of care was implemented; we added three additional questions in the form of a Likert scale ranging from 1 = disagree to 3 = agree at the 3 endpoints T0, T1 and T2.

1. There is a clear philosophical nursing perspective in the institution where I work.

2. Nursing care is based on a nursing model rather than on a biomedical model.

3. The care I give is congruent with my personal values.

Two focus groups were conducted in June 2019 by two external members of the project (one facilitated the exchanges, inviting each participant to express themselves following the interview guide prepared in advance, and the other had an observational role and was responsible for time and the creation of a favourable environment as well as for recording) [35] to gain a deeper understanding of the ECN’s perspective towards the SBNH and their experience regarding the implementation process. The questions addressed the changes perceived by the ECNs since the introduction of SBNH, the effects on the families, the network and their wishes for the future to take their ownership of the model. Participation in the focus groups was voluntary. The first focus group was conducted with eight nurses who received supervision from the trainer. The second focus group was composed of ten ECNs who did not received this supervision. An interview guide was used during the focus groups.

### Study analyses

#### Quantitative data

Descriptive analyses were used to describe the characteristic of the sample. The Shapiro Wilk test was used at each time point to test the normality of the mean scores of competencies. Changes of perceived competencies over time were tested using the Kruskal Wallis test. To compare the frequency of perceived competencies’ utilization between time points (T0-T1-T2) Chi-squared test were used.

A logistic regression was used to test the impact of age, work experience and work rate on the ECN’s competencies improvement between T0 and T2. Improvement between T0 and T2 was dichotomized as followed for the analysis (0 = no improvement; 1 = improvement), *P*-value was fixed at 0.05. Analyses were performed using Stata IC 16 [36].

#### Qualitative data

Focus groups were recorded and then transcribed verbatim. Thematic analysis was used to analyze the verbatim transcripts of the focus groups, following the steps described by Miles & Huberman [37]: data reduction, data display, conclusions drawing and verification.

Independent, inductive thematic analyses were carried out by two external experts using MAXQDA analytics pro-2020. Data were coded and analyzed by two coders separately. Periodic meetings between coders were held to resolve discrepancies and reach consensus at each step of the analysis. This allowed the research team to create a codebook. After pooling, the two coders and an expert discussed and agreed upon the identified themes and subthemes [37]. In order to ensure the rigor and credibility of the qualitative data, this study used a focus group guide, investigator triangulation and two interviewers who were independent of the project. Finally, an expert group was created for verification of the themes and subthemes.

## Results

### Quantitative data

Questionnaires were sent to every nurse at the institution (*N* = 61) regardless of when they started working at the institution or whether they had participated at T0. As this study was aimed at implementation, all opinions were considered to capture the real trend. During the study, 109 questionnaires were completed by 61 ECN. Eighteen ECN did not participate at T0, because some started their employment after the collection at T0, others did not take the time to answer or did not wish to participate at the beginning and changed their mind during the project. Fifteen ECN participated at T0 only. Questionnaires from nurses who did not participated at T0 or only participated at T0 were excluded from the pre-post analysis. Therefore, the pre-post analysis included questionnaires from 28 ECN (73 questionnaires) who participated at T0 and at least one other time point. A total of 17 ECNs completed the questionnaire at all time points.

### Participant characteristics

The largest proportion of ECN were aged > 50 years (23/43, 53.5%), with hospital work experience ranging between 5 to 10 years (15/43, 34.9%) or over 10 years (17/43, 39.5%). Many nurses (14/43, 32.6%) had occupied their actual post for the previous 15 years. They mainly worked part time (See Table 2).

**Table 2:** Sociodemographic characteristics of participants

Characteristic	T0 (*n* = 43)
	*n* (%)
**Age**	
30–39 years	9 (20.9)
40–49 years	11 (25.6)
50 years and over	16 (37.2)
Over 60 years	7 (16.3)
**Level of nursing degree**	
Generalist nurse, level II	21 (48.8)
Maternal and paediatric hygiene nurse	14 (32.6)
Other	5 (11.6)
Higher degree / Bachelor	3 (7.0)
**Hospital Experience**	
None	1 (2.3)
Between 3 and 5 years	10 (23.3)
Between 5 and 10 years	15 (34.9)
Over 10 years	17 (39.5)
**Working experience at actual post**	
Between 0 and 5 years	14 (32.6)
Between 6 and 10 years	11 (25.6)
Between 11 and 15 years	4 (9.3)
Over 15 years	14 (32.6)
**Work rate at actual post**	
Between 10 and 20%	8 (18.6)
Between 30 and 50%	13 (30.2)
Between 60 and 80%	22 (51.2)

### Perceived competencies at T0

Medians (Med) and standard deviation (SD) were calculated for each competency and for the NCS global score in order to obtain a picture of nurses’ perceptions of their competencies before the intervention (See Table 3).

**Table 3:** Perceived competencies at T0

	*Med*	*SD*
	*(n = 43)*	
Helping role	73.3	12.3
Teaching-coaching	70.6	14.4
Diagnostic functions	69.7	15.5
Managing situations	70.6	12.9
Therapeutic interventions	67.5	14.9
Ensuring quality	65.0	18.3
Work role	70.1	12.6
NCS global score	68.2	12.8

Nurses perceived themselves as more competent in the “Helping role” competency and less competent in “Ensuring quality”.

### Evolution over time of ECN’s positions in relation to the vision of care

ECN’s agreement with the three affirmations about the vision of care increased over time, from T0 to T2 (See Table 4).

**Table 4:** Evolution of ECN’s positions in relation to the vision of care

	T0 (*n* = 28) *n* (%)	T1 (*n* = 25) *n* (%)	T2 (*n* = 20) *n* (%)
**There is a is a clear philosophical nursing perspective in the institution where I work**			
Disagree	1 (3.6)	1 (4.0)	–
Neither disagree, nor agree	8 (28.6)	5 (20.0)	1 (5.0)
Agree	19 (67.9)	19 (76.0)	19 (95.0)
**Nursing care is based on a nursing model rather than a biomedical model**			
Disagree	2 (7.1)	2 (8.0)	–
Neither disagree, nor agree	9 (32.1)	4 (16.0)	3 (15.0)
Agree	17 (60.7)	19 (76.0)	17 (85.0)
**The care I give is congruent with my personal values**			
Disagree	1 (3.6)	3 (12.0)	–
Neither disagree, nor agree	2 (7.1)	1 (4.0)	–
Agree	25 (89.3)	21 (84.0)	20 (100.0)

### Pre-post analyses

#### Frequency of action

Frequency of action has increased between T0 and T2 for all competencies except for “teaching-coaching”. No significant differences were found (See Table 5).

**Table 5:** Frequency of action « occasionally » or « very often » for each domain

	T0 (*n* = 28)	T1 (*n* = 28)	T2 (*n* = 28)		
	Freq	Freq	Freq	**χ2**	***p*-value**
Helping role	85.2%	86.9%	92.9%	8.157	0.227
Teaching-coaching	71.9%	74.0%	71.9%	8.305	0.217
Diagnostic functions	63.8%	69.1%	66.4%	8.584	0.198
Managing situations	53.6%	57.5%	56.9%	4.699	0.583
Therapeutic interventions	56.8%	57.2%	64.5%	7.810	0.252
Ensuring quality	64.9%	64.7%	75.0%	8.247	0.221
Work role	66.2%	69.5%	68.7%	2.845	0.828

#### Evolutions of perceived competencies over time

**Fig. 2:**
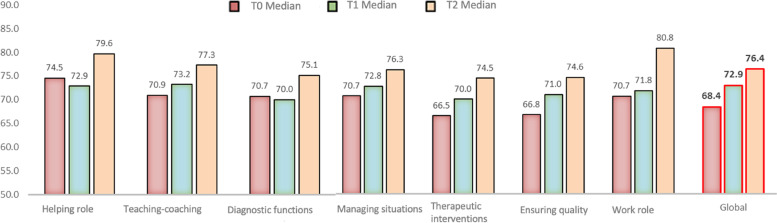
Evolution of perceived competencies over time

The median of all the perceived competencies increased over time, except for the “helping role” and “diagnostic functions”. These two competencies decreased at T1 and then increased at T2 (See Fig. 2).

#### The change in the degree of perceived competencies over time

The Kruskal Wallis test showed no significant differences in the degree of perceived competencies over time, except for the “helping role”, “therapeutic interventions” and “work role”, which increased over time (See Table 6).

**Table 6:** Change in the degree of perceived competencies over time (n = 28)

	*χ*^2^	*p*-value
Helping role	6.953	0.031*
Teaching – coaching	4.321	0.115
Diagnostic functions	3.353	0.187
Managing situations	2.149	0.342
Therapeutic interventions	6.326	0.042*
Ensuring quality	4.461	0.107
Work role	7.107	0.029*
Global score	5.578	0.062

*p < 0.05

#### Competencies score: logistic regression – confounding factors

A logistic regression was used to test the association of age, working experience/rate and their perceived level of competencies between T0 and the last test point (either T1 or T2). Age and length of work experience in the institution lowered the probability of improving their “teaching-coaching” competencies (See Table 7).

**Table 7:** Logistic regression of competencies

	*OR*	*p*-value	95% confidence interval	
**Helping role**				
Age	0.439	0.095	0.167	1.154
Work rate	0.778	0.743	0.173	3.493
Experience in the institution	0.750	0.359	0.405	1.388
**Teaching– coaching**				
Age	0.092	0.020*	0.013	0.682
Work rate	0.582	0.507	0.118	2.880
Experience in the institution	0.213	0.005*	0.072	0.624
**Diagnostic functions**				
Age	0.414	0.101	0.144	1.189
Work rate	1.125	0.885	0.229	5.537
Experience in the institution	0.764	0.418	0.399	1.464
**Managing situations**				
Age	0.841	0.702	0.346	2.045
Work rate	1.125	0.885	0.229	5.537
Experience in the institution	1.055	0.870	0.555	2.003
**Therapeutic interventions**				
Age	0.680	0.411	0.271	1.070
Work rate	2.222	0.344	0.426	11.603
Experience in the institution	0.683	0.260	0.352	1.325
**Ensuring quality**				
Age	0.540	0.213	0.204	1.425
Work rate	1.125	0.885	0.226	5.537
Experience in the institution	0.764	0.418	0.399	1.464
**Work role**				
Age	0.390	0.078	0.137	1.112
Work rate	1.500	0.612	0.313	7.186
Experience in the institution	0.816	0.526	0.435	1.530
**Global score**				
Age	0.192	0.024*	0.046	0.807
Work rate	1.125	0.885	0.229	5.537
Experience in the institution	0.537	0.084	0.265	1.086

*p < 0.05

### Qualitative data

**Fig. 3:**
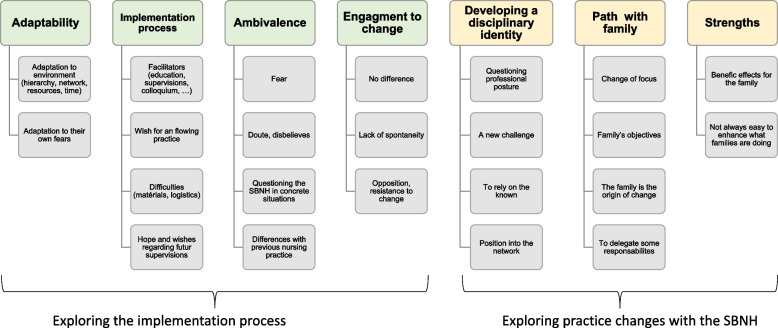
Themes and subthemes from the ECN’ experiences of the implementation process and their change in practice

Analysis of the two focus groups identified seven themes and 23 subthemes, illustrating how nurses experienced the implementation and the daily utilization of the SBNH. The themes “adaptability”, “implementation process”, “ambivalence” and “resistance to change” were related to implementation. The daily use of the philosophy was reflected by “professional posture/ disciplinary identity”, “the path with the family” and “strengths” (See Fig. 3).

After identifying the themes and sub-themes, we found that the same themes emerged in both groups (with and without supervision). A consensus was found on the changes with the new philosophy and on the important themes to consider in the implementation process. We were able to highlight the importance of supervision in the implementation process, which was strongly noted in both groups.

### Exploration of the implementation process

#### Adaptability

This theme refers to “the capacity to adapt to new environments or situations” [38]. ECN had to demonstrate this on several levels throughout the SBNH implementation process. They had to adapt to hierarchy, professional networks, instruments and time, but also to cultural differences. Participant 12: *“I think our activity requires a lot of adaptation and sensibility. You have to be flexible.”*

Furthermore, they had to adapt to their fears and possible feelings of helplessness. Participant 20: “*… When they (the family) have precise expectations on precise subjects, for me, it is complicated to highlight their strengths and mobilise their resources.”*

#### Implementation process

According to the ECN, the implementation process was facilitated by the theory provided during the training day and the supervision sessions and by the regional colloquium with the respective head nurses. In particular, participants reported that exchanges with colleagues very helpful.

Participant 17: “The *fact that we could discuss during supervisions sometimes, because we tried to keep track*.”

The ECN encountered some difficulties in the implementation process due to a lack of resources. Those who had not already received supervision sessions with the nursing sciences specialist hoped that this supervision would be helpful. Participant 18: “*I believe that we will benefit from the supervision, because for me, it will really help me, so that it become automatic.*”

#### Ambivalence

Some ECN expressed doubts or fears that SBNH could lead to a one-size-fits-all approach, which could be dogmatic, lead to a sense of confinement and not meet parent’s expectations. Participant 13: “*I’m sometimes a little afraid with Gottlieb that there’s something about going into religion, I tell you frankly*.”

They wondered if this approach could be used when families encountered concrete practical problems. They also expressed questions regarding their previous practice, without this approach. Participant 14: *“… Before, I had the impression that I was focusing more on the parents’ preoccupations rather than mine.”*

#### Engagement to change

Some ECN reported that they did not see significant differences between their previous and their practice using SBNH. Participant 2: “*I agree with you, I say to myself that it is often necessary to encourage, congratulate parents; these are things I am sure I used to do before*.” They also reported a possible lack of spontaneity with families and difficulties in applying this theory for certain types of families. Some ECN expressed resistance to change. Participant 3: “*It is not a panacea, but just as I wouldn’t accept being told that I have to increase my number of first visits, I wouldn’t accept being told that I have to do Gottlieb all the time and that I have to make a report in my file according to Gottlieb, otherwise I will get bad points.*”

### Exploration of practice change with the SBNH approach

#### Developing a disciplinary identity

The ECN questioned their professional posture and their disciplinary anchorage. Indeed, they noted that SBNH taught them to approach parents and families with a certain humility, and it added a new arrow to their quiver. They felt a change in the understanding of their position, allowing them to delegate some of their responsibilities to parents. Participant 5: “*It has allowed me sometimes to say no, now I let go because I am looking for the other person’s competencies so somewhere along the line I take a weight off my shoulders.*”

Some ECN took this change as a challenge/opportunity to question their routine. Participant 19: “… *Because after a number of years, you are in a certain routine*.” Despite this, they emphasised the importance of building on their past experiences. They also questioned their posture and place inside their professional network, without giving them any additional self-confidence.

#### Path with families

ECN changed their priorities, focusing less on need and more on the families’ objectives. Participant 15: *“The word ‘needs’ is a word that I tried to erase from my vocabulary.”* They were aware that it was the family who would initiate the desired changes, and, in this sense, they emphasised the importance of being genuinely present with the patient. Participant 4: “*Well, when we are less in a hurry, it is when the family is ready for change, if we are less in a hurry, well, then the family moves on and there is less pressure.*”

They judged this approach as positive for the families and felt relief at being able to delegate some of their responsibilities to the families, Participant 12: “*I am learning to identify their resources and I say to myself that they have some and I feel relieved*.”

The ECN agreed that SBNH was effective for a long-term follow-up but were doubtful about using it when families needed solutions. They thought families would not be able to handle having no solutions (for example when they have specific questions about diseases). Participant 19: “*It’s true that if I were the mother, I think it would annoy me if every time I asked a question it was returned to me; I wouldn’t stand it”.*

#### Strengths

This theme is related to the families’ strengths that the ECN drew out and the benefits the families derived from them. Participant 19: “*We can feel that in moments like this, the huge effect of highlighting was going well.”* They, however, mentioned that this process was not always easy.

## Discussion

This mixed-method, pre-post intervention study highlighted that ECN considered themselves more competent 18 months after the intervention. The frequency of use of the competencies also increased after the intervention. The ages of the ECN and the time they had spent at their actual post influenced their perception of their “teaching-coaching” competency. It also appears that the ECN gradually adopted a different posture within the care network. Indeed, some professionals noticed a difference. Families were more likely to thank the ECN for their support and guidance rather than for their advice.

### Sociodemographic characteristics of the sample

The majority of the ECN were over 50 years old and had previous hospital working experience, half of them occupied their actual posts for more than 15 years and most of them worked part time. The characteristics of our sample were similar to those of other international ECN samples in the literature [10, 39, 40]. In accordance with another study, except for age and time at their actual post, these characteristics had no influence on the frequency of action [41]. Indeed, in our sample, the longer they occupied their actual post or the older they were, the less their “teaching-coaching” competency improved. This is not in line with the findings of other studies, which found that the older and more experienced the nurses were, the higher the frequency of action was [30, 42–44]. This also seemed to be the case for nurses working full time who had more advanced degrees [44]. This could be explained by the fact that in our context, the frequency of action for “teaching-coaching” was already high before the intervention and the majority of nurses are working part-time. Furthermore, in their working context, they are fewer opportunities to coach new nurses.

### Evolution over time of ECN’s positions on the philosophy of care

Before the intervention, most nurses were convinced that they needed a clear philosophy of care in their practice (67.9%) and that the care they provided aligned with their values (89.3%). This is also supported by the qualitative results for the theme “engagement to change”. Nurses stated that they already held this philosophy as a basic tenant of their practice and that they did not see significant differences with what they had done before. The percentage of ECN who agreed with these questions increased from T0 to T2, supporting Gottlieb’s view that “SBNH is about discovering, developing and amplifying strengths” [16].

### Perceived competencies and frequency of action

Quantitative analyses showed that ECN perceived themselves as more competent over time in their helping role, in therapeutic intervention and in their work role. In our study, ECN considered themselves most competent in their helping role and least competent in ensuring quality. This aligns with two cross-sectional studies [30, 43].

No changes were found in the degree of perceived competencies for the following competency categories: “teaching-coaching”, “diagnostic function”, “managing situations” and “ensuring quality”. For diagnostic function, the frequency of action decreased from T0 to T1 and increased from T1 to T2. One possible explanation for the lack of perceived increase over time is that ECN were most likely already competent in this domain before the intervention. Indeed, the focus of the framework that guided nurses’ practice before the implementation of SBNH was on identifying needs, strengths and potential difficulties that might induce health problems. In this framework, the nurse would find adapted solutions in collaboration with families [9]. This echoed Gottlieb’s assumption that SBNH focuses on discovering, updating, and growing strengths.

### Qualitative themes

**“Adaptability”** theme is an essential component of the helping role. Indeed, they need to adapt to a shift in their role [29]. Nurses also need to adapt to various situations and family contexts. According to the World Health Organization, “health professionals must be able to adapt to cultural variations and values, as well as attitudes to the different health problems of populations.” (page 23) [45]. Questioning what the main strengths are and how to reinforce them and which new should be developed is One characteristic of SBNH that reinforce these findings, underlining the importance of adaptability to family needs, is the questioning of what main strengths are, how to reinforce them and which should be developed.

In the theme “Implementation process”, nurses mentioned that the process of implementing SBNH was facilitated by exchanges with colleagues and supervisors in practice workshops. This echoes the SBNH assumption that humans are wired for strengths and organizations have inherent strengths; they are necessary for survival and growth [16]. In other studies, nurses reported that continuous support, supervision, and education were essential for implementing and sustaining a strength-based approach [28, 29, 46].

The theme “ambivalence” reflected the scepticism of the ECN regarding the use of SBNH. Other studies also reported nurses’ ambivalence regarding the implementation of such an approach [22, 46], especially when families were experiencing urgent difficulties that could lead to the endangerment of children [22]. However, Gottlieb mentioned that SBNH does not ignore problems and does not pretend that deficits and weaknesses do not exist [13]. The utilization of SBNH should not be limited, and it is applicable in every situation encountered in ECN’s daily practice. Nurses also questioned their adequacy with the approach in the theme “engagement to change”. Gottlieb’s assumption that nurses should question what principles are guiding their practice supports this finding [7]. Some showed resistance to change. This aligned with other studies that found that some nurses were reluctant to change their practice [29, 46].

ECN recognised their limits and delegated some of their responsibilities to the family, as shown in the theme “developing a disciplinary identity”. They also emphasized developing multidisciplinary team care and networking. According to Gottlieb, when nurses are aware of their values, attitudes and beliefs, they can take control of their practice and carry out their professional tasks in a way that is more consistent with what they consider important [7]. Their positions on the approach also changed, as noted in other studies in which nurses questioned their routine and changed the understanding of their role [29, 46].

ECN mentioned that discussing questions with families instead of immediately giving them an answer was beneficial. In the theme “path with families”, ECN underlined the importance of being authentically present and that families were at the origin of wanted changes. Other studies have reported the importance of the relationship between the ECN and parents as a way to engage and give control to parents [28, 29]. These characteristics are related to the importance SBNH places on the discovery and amplification of families’ strengths [16]. The notion of systematic evaluation of the families’ satisfaction is also mentioned, a concept that is related to the last step of Gottlieb’s nursing interview: revision [7].

### Study strengths and limitation

This study has several strengths and limitations. One major strength is the innovation represented by the implementation of SBNH in an early childhood nursing context – more specifically, its documentation and evaluation. Indeed, the implementation followed a specific process at every stage, from choosing the model to implementing its daily use. The utilisation of a validated questionnaire allowed rigorous documentation of the results. The mixed-method design produced a deeper understanding of the nurses’ experience.

A major limitation of this study is the relatively small sample, which impedes the generalizability of the finding. Furthermore, the loss of participants (e.g.: to retirement) before the follow-up data was collected represents a significant limitation that is a common difficulty in longitudinal studies [47]. The results of this monocentric study should be interpreted in the study’s regional context, where nurses’ values and beliefs might be different from those of nurses in other contexts. The use of a self-reported questionnaire to rate their level of expertise might have induced a social-desirability bias [47]. Furthermore, the questionnaire is long, which may have discouraged some nurses from answering, explaining the missing data. Another limitation is that we could not assess readiness for change in the institution as the Organizational Readiness for Change Assessment questionnaire [48] has not been adapted to the context of home care.

### Implications for practice, research and education

The use of conceptual care models or theories to guide the nursing practice has been proven to add value. In particular, it allows nurses to express what they do and why they do it [49]. This was confirmed by the nurses’ change in point of view regarding the philosophy of care.

Questioning one’s practice is an essential process for ensuring quality of care. Similarly, being aware of good practice guidelines and the importance of research helps to implement the insights of that research and promotes real changes in practice through adaptations to real life.

This study showed ECN’s perceived competencies changed over time after the introduction of SBNH into practice. Regardless of age, years of practice or time at their current job, the ECN questioned their routine and adapted their practice based on SBNH.

From the patients’ point of view, theory-guided practice affected their quality of life, self-efficacy and stress [49]. For families, this approach allowed a change of vision to one with a resource-centred approach, a benefit that should be further explored in another study.

Little research has addressed the implementation of a model of care or theories of care in clinical practice, particularly in the early childhood setting [5]. Very few published studies specify the theoretical framework used, making it impossible to compare the added value in different contexts. Further research that clearly specifies the theoretical framework and context should be carried out. Such research could show the importance of basing practice on a common care model at a large scale and the primary importance of nursing in our health care system. Regarding students and new collaborative mentorship, SBNH is aware that strengths are necessary for survival, growth, fulfilment and relationships [16]. This is also supported by the literature [19].

Thus, it is necessary to continue teaching SBNH in pre- and post-graduate training. This will make nurses aware of the strength of sharing a common language within the same health care structure and of the profession’s crucial influence within the health care system.

## Conclusion

ECN’s profession is demanding and requires a sound working experience. They must be prepared to handle many situations and must manage the unexpected. In challenging situations, a strength-based approach allows the beneficiaries (here, the families) to be more autonomous by developing their own competencies enabling this requires knowledge of the family as well as strong collaboration with them. Strengthening nursing care with this approach not only allows better utilization of nursing skills but also encourages greater involvement with the families, thus developing their own capacities.

This study documented the implementation process of an approach perceived as effective for nursing competencies in a specific context. It shows that most of the competencies and their frequency of actions increased in the process. As the implementation process was well received by nurses, it would be interesting to promote its dissemination into other settings.

## Data Availability

The transcripts that generated the qualitative data for analysis in this study are not publicly available to protect the participants’ anonymity. The quantitative datasets are available from the corresponding author on reasonable request.

## Abbreviations

ECN: Early Childhood Nurses
SBNH: Strengths-Based Nursing and Healthcare
NCS: Nurse Competence Scale
